# The Complete Chloroplast Genome Sequence of the Medicinal Moss *Rhodobryum giganteum* (Bryaceae, Bryophyta): Comparative Genomics and Phylogenetic Analyses

**DOI:** 10.3390/genes15070900

**Published:** 2024-07-10

**Authors:** Zhengyuan Shen, Qin Liu, Jiewei Hao, Sheng Bi, Yezhen Fu, Lina Zhang

**Affiliations:** 1 Center for Eco-Environment Restoration Engineering of Hainan Province, School of Ecology, Hainan University, Haikou 570228, China; szy874140673@outlook.com (Z.S.);; 2 Guangxi Key Laboratory of Agricultural Resources Chemistry and Biotechnology, Yulin Normal University, Yulin 537000, China; 3 Bawangling Branch of Hainan Tropical Rainforest National Park Administration, Changjiang 572700, China; 4 Ministry of Education Key Laboratory of Genetics and Germplasm Innovation of Tropical Special Forest Trees and Ornamental Plants, Hainan University, Haikou 570228, China

**Keywords:** *Rhodobryum giganteum*, chloroplast genome, comparative analysis, Bryales, phylogenetic analysis

## Abstract

*Rhodobryum giganteum* (Bryaceae, Bryophyta), a rare medicinal bryophyte, is valued for its cardiovascular therapeutic properties in traditional Chinese medicine. This study presents the first complete chloroplast genome sequence of *R. giganteum*, including its assembly and annotation. The circular chloroplast genome of *R. giganteum* is 124,315 bp in length, displaying a typical quadripartite structure with 128 genes: 83 protein-coding genes, 37 tRNAs, and 8 rRNAs. Analyses of codon usage bias, repetitive sequences, and simple sequence repeats (SSRs) revealed an A/U-ending codon preference, 96 repetitive sequences, and 385 SSRs in the *R. giganteum* chloroplast genome. Nucleotide diversity analysis identified 10 high mutational hotspots. Ka/Ks ratio analysis suggested potential positive selection in *rpl20*, *rps18*, *petG*, and *psbM* genes. Phylogenetic analysis of whole chloroplast genomes from 38 moss species positioned *R. giganteum* within Bryales, closely related to *Rhodobryum laxelimbatum*. This study augments the chloroplast genomic data for Bryales and provides a foundation for molecular marker development and genetic diversity analyses in medicinal bryophytes.

## 1. Introduction

*Rhodobryum giganteum*, a species within the genus *Rhodobryum* (Bryophyta: Bryaceae), commonly inhabits moderately high-altitude tropical and subtropical forests across Asia, Papua New Guinea, Hawaii, and Madagascar. This species has been documented colonizing diverse substrates such as tree trunks, wet banks, rocks, decaying wood, humus, and shaded ground [1]. In China specifically, it mainly occurs in the regions south of the Yangtze River, typically thriving in understory grasses, on moist humus, or on thin soil covering damp rocky surfaces [2]. Despite its small size and simple morphology, *R. giganteum*, along with other bryophytes, plays a vital role in water and soil conservation, nutrient cycling, ecosystem restoration, serving as ecological indicators, and providing microhabitats for various small invertebrates, thus contributing to local biodiversity [3]. In some areas, it also serves as an indicator species for air quality due to its sensitivity to atmospheric pollutants [4]. Moreover, *R. giganteum*, known as ‘Huixincao’ in traditional Chinese medicine, has been utilized for centuries to treat various conditions, including fever, neurasthenia, and psychosis [5,6]. According to the compilations of Chinese herbal medicine [7,8], *R. giganteum* is believed to nourish the heart, calm the mind, clear the liver, and improve vision. It has been traditionally used for treating conditions such as palpitations, myocarditis, coronary heart disease, neurasthenia, psychosis, red eyes, and wounds, and is also believed to lower blood viscosity and have anti-atherosclerotic effects. These diverse applications of *R. giganteum* in traditional medicine demonstrate its multi-faceted therapeutic capabilities. Recent studies have further highlighted *R. giganteum*’s therapeutic potential for cardiovascular diseases and hypertension, with no reported toxicity in humans [9]. Despite extensive research on *R. giganteum*’s pharmacologically active components [10,11,12,13,14], genomic studies remain limited. Given its rich traditional uses, analyzing the chloroplast genome of *R*. *giganteum* can offer valuable insights into the genetic basis of its medicinal properties. This genomic information, coupled with recent advancements in bryophyte cultivation techniques, can support genetic breeding efforts and guide future pharmacological and therapeutic applications.

The chloroplast genome, characterized by uniparental inheritance, structural conservation, and moderate evolution rate [15,16], serves multiple functions, including molecular marker development, barcode identification, and phylogeographic analysis [17]. High-throughput sequencing has facilitated extensive chloroplast genome characterization, enhancing plant phylogenetics and molecular classification studies [18]. Mosses, representing an early divergent lineage in land plant evolution [19], remain understudied due to the limited availability of complete chloroplast genomes, impeding a comprehensive understanding of moss genome evolution [20].

This study presents the chloroplast genome of *R. giganteum* and compares it with seven other Bryales species. Our objectives were to elucidate the structural features of the chloroplast genome of *R. giganteum* and to construct a genome-wide phylogeny of moss chloroplasts. The complete sequence of *R. giganteum* provides a foundation for upcoming studies on phylogeny, taxonomy, species identification, population genetics, genetic breeding, and conservation strategies of this medicinally significant moss.

## 2. Materials and Methods

### 2.1. Sample Gathering, DNA Extraction, and Sequencing

Specimens of *Rhodobryum giganteum* were collected from the understory humus soil in the tropical cloud forest (TCF) of Bawangling, Changjiang County, Hainan Province, China (109°12′40.49″ E, 19°5′12.00″ N, 1370 m) in July 2023. Six individual plants were collected, and the voucher specimen was deposited in the Hainan University Herbarium (HUTB). Samples were carefully rinsed with ddH_2_O, flash-frozen in liquid nitrogen, and stored at −80 °C prior to DNA extraction. For sequencing, fresh plant material was submitted to Genepioneer Biotechnologies (Nanjing, China).

Genomic DNA was extracted from 0.1 g of *R. giganteum* tissue using the cetyl trimethyl ammonium bromide (CTAB) method. The CTAB method was chosen for DNA extraction due to its effectiveness in isolating high-quality DNA from plant tissues rich in polysaccharides and secondary metabolites, which are common in bryophytes. Whole genomic DNA was sequenced using paired-end (PE) sequencing on the Illumina NovaSeq 6000 platform with 150 bp read lengths. A total of 25.6 million raw reads were obtained, and the raw sequences were further used for quality control by fastp v0.20.0 [21]. The default settings of fastp include automatic adapter trimming of sequencing junctions in reads and primer sequences, low-quality base trimming (with a quality threshold less than 5), and read filtering (average quality value less than Q5). After cleaning, 25.4 million high-quality paired-end reads were retained and used for subsequent analysis.

### 2.2. Chloroplast Genome Assembly and Annotation

The chloroplast genome assembly process began with the use of Bowtie2 v2.3.5 [22] to align the cleaned reads against a database of known moss chloroplast genomes, enriching for chloroplast-derived sequences. These aligned reads (0.56 million) were assembled de novo using SPAdes v3.10.1 software [23] with k-mer sizes of 55, 87, and 121. The assembly process resulted in a total of 3 contigs. These chloroplast contigs were organized into scaffolds using SSPACE v2.0 [24], and any remaining gaps were filled using Gapfiller v2.1.1 [25]. The final assembly consisted of 1 scaffold with a total length of 124,315 bp, representing the complete chloroplast genome. The average depth of the final chloroplast genome assembly was 1642 x. To improve annotation accuracy, a two-step process was employed. Initially, Prodigal v2.6.3 (https://github.com/hyattpd/Prodigal, accessed on 10 August 2023) was utilized for predicting chloroplast coding sequences (CDS) due to its high sensitivity, Hmmer v3.1b2 (http://www.hmmer.org/, accessed on 10 August 2023) for ribosomal RNA (rRNA), and Aragorn v1.2.38 (http://www.ansikte.se/ARAGORN/, accessed on 10 August 2023) for transfer RNA (tRNA) identification. Subsequently, an alternative annotation was generated based on related species data from NCBI (https://www.ncbi.nlm.nih.gov/, accessed on 10 August 2023) and validated with BLAST v2.6 (https://blast.ncbi.nlm.nih.gov/Blast.cgi, accessed on 10 August 2023) to ensure comprehensive gene identification. Erroneous and redundant annotations were manually removed based on the results of these two annotations. Intron/exon boundaries were confirmed manually using sequences from closely related species as a reference. The LSC (large single-copy) regions, SSC (small single-copy) regions, and IR (inverted repeat) regions of the chloroplast genome were annotated with the Repeats Finder plugin in Geneious Prime 2022.2.2 [26], chosen for its user-friendly interface and powerful analysis. Additionally, tRNA gene identification was performed using the tRNAscan-SE v2.0.5 [27]. Finally, the circular genomic map was drawn using OGDRAW [28].

### 2.3. Codon Usage Bias, Repeat Sequence, and SSRs Analyses

Relative synonymous codon usage (RSCU) compares observed occurrences with expected frequencies, where values > 1 indicate positive bias, <1 negative bias, and 1 no bias [29]. CDS sequences (≥300 bp) were extracted from the chloroplast genome of *R. giganteum* using Geneious Prime v2022.2.2 [30]. RSCU was calculated using CodonW 1.4.2 [31]. Simple sequence repeats (SSRs) were identified using MISA [32], with minimum repeat thresholds of 8, 5, 3, 3, 3, and 3 for mono-, di-, tri-, tetra-, penta-, and hexanucleotides, respectively [33]. REPuter annotated repetitive sequences in the chloroplast, with parameter offset to Hamming Distance = 3 and Minimum Repeat Size = 30 [34].

### 2.4. Comparative Analysis of the Chloroplast Genome

To investigate the divergence between the genome sequence of *R. giganteum* and other Bryales species, we obtained genome sequences of 7 other bryophyte species available in the GenBank database. These species include *Rhodobryum laxelimbatum* (NC_056918.1), *Bryum argenteum* (NC_058542), *Anomobryum gemmigerum* (NC_069305), *Mnium marginatum* (NC_054293.1), *Plagiomnium acutum* (MZ297476), *Pohlia cruda* (NC_056136) and *Pohlia nutans* (NC_045869). Comparative analysis was conducted using mVISTA to identify highly variable regions [35]. IR, SSC, and LSC boundary regions were visualized using CPJSdraw software [36]. Nucleotide variability (Pi) was assessed using DnaSP v6 (window length: 600 bp, step size: 200 bp) [37]. These values were calculated using KaKs_calculator 2.0 with *R. giganteum* as a reference [38], employing the YN method and Transl_Table11 for protein-coding genes (PCGs) [39].

### 2.5. Phylogenetic Analysis

To investigate the phylogeny of *R. giganteum*, a phylogenetic tree was created by analyzing the chloroplast genome sequences of 38 moss species, with *Takakia lepidozioides* serving as an outgroup. Whole genome sequences were aligned through MAFFT [40] in Geneious Prime v2022.2.2, and the best model (TVM+F+R5) was determined by ModelFinder [41]. Maximum-likelihood (ML) trees were generated with 1000 bootstrap replicates using IQ-TREE [42], and the final trees were visualized and edited through the iTOL website (https://itol.embl.de/, accessed on 1 April 2024).

## 3. Results

### 3.1. Chloroplast Genome Characteristics of R. giganteum

**Figure 1:**
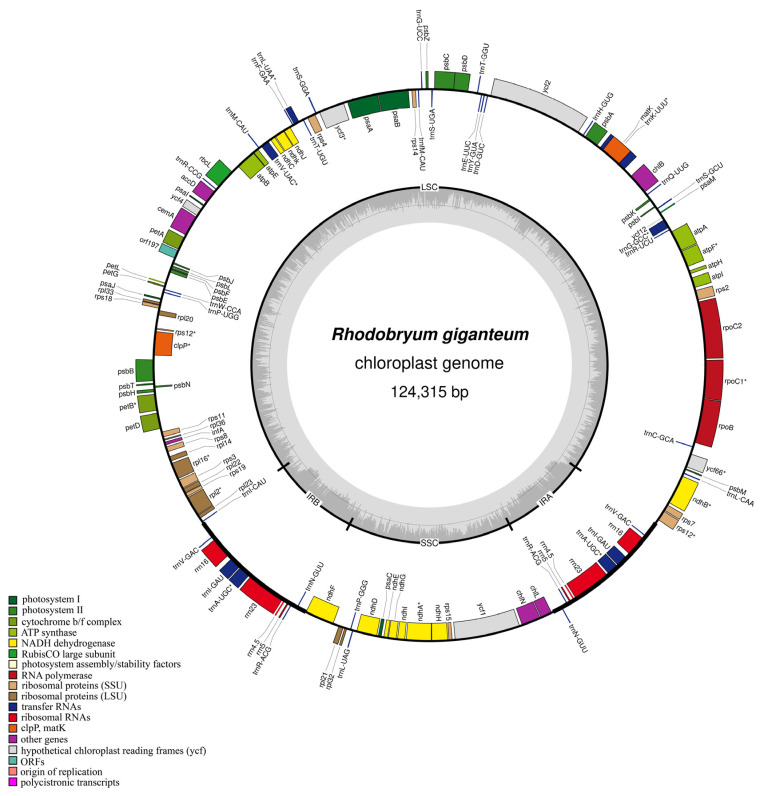
Chloroplast genome map of *R. giganteum*. The different colors represent genes of different functional groups. The genes drawn inside are transcribed clockwise, while the outside genes are transcribed counterclockwise.

The chloroplast genome of *R. giganteum* exhibits a typical quadripartite structure, totaling 124,315 bp (refer to Table 1 and Figure 1). It comprises a pair of inverted repeat (IR) regions (IRa/IRb, 9481 bp each), separated by large single-copy (LSC, 86,261 bp) and small single-copy (SSC, 18,371 bp) regions. The overall GC content is 30.17%, with variations among IRa/IRb (44.1%), LSC (27.6%), and SSC (27.6%) regions (Table 1). The genome encodes 128 functional genes: 83 protein-coding genes, 37 tRNA genes, and 8 rRNA genes (Table S1), categorized into photosynthesis-related genes, self-replication-related genes, other genes, and genes with unknown function. Fifteen genes contain a single intron (*ndhA*, *ndhB*, *petB*, *petD*, *atpF*, *rpl16*, *rpl2*, *rpoC1*, *ycf66*, *trnA*-*UGC*, *trnG*-*GCC*, *trnI*-*GAU*, *trnK*-*UUU*, *trnL*-*UAA*, and *trnV*-*UAC*), while *clpP* and *ycf3* each possess two introns (Table S1).

### 3.2. Codon Usage Bias

**Figure 2:**
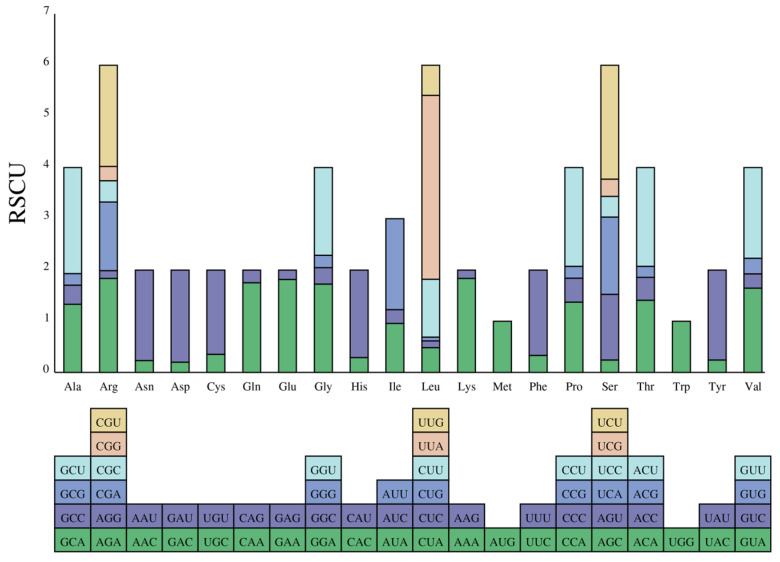
RSCU values of 20 amino acids in the CDS of the *R. giganteum* chloroplast genome. Boxes below the graphs represent all codons encoding each amino acid, with the colors of the histograms corresponding to the colors of the codons.

Codon usage analysis in the *R. giganteum* chloroplast genome (Figure 2, Table S2) revealed Leu as the most abundant amino acid (2172 occurrences), followed by Ile (1895), while Ter showed the lowest frequency (52). RSCU values were highest for the Leu codon UUA (3.59) and lowest for the Leu codon CUG (0.07). Among the 29 codons with RSCU > 1, most ended with A/U, indicating a bias. Only AUG (Met) and UGG (Trp) showed no bias (RSCU = 1) (Table S2).

### 3.3. Repeat Sequence and SSRs Analysis

**Figure 3:**
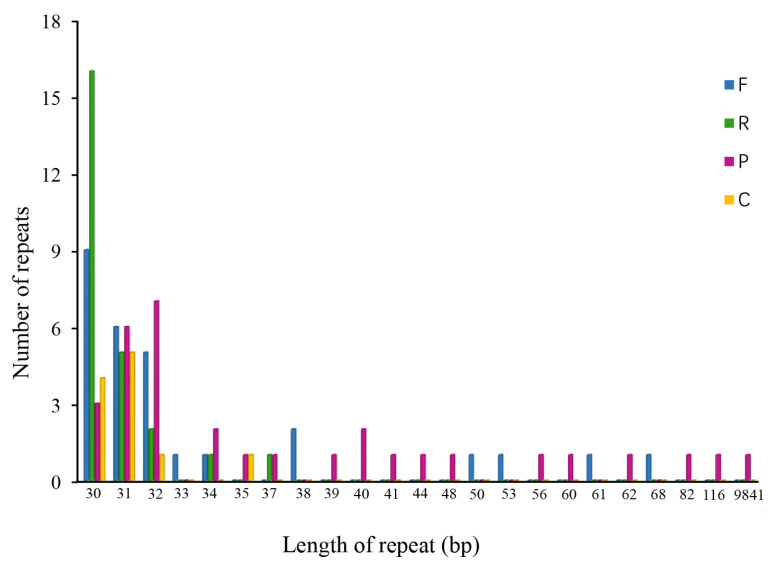
Numbers of repetitive sequences in the complete chloroplast genome of *R. giganteum*. Forward repetition was abbreviated as F, palindromic repetition was abbreviated as P, reverse repetition was abbreviated as R, and complementary repetition was abbreviated as C.

**Figure 4:**
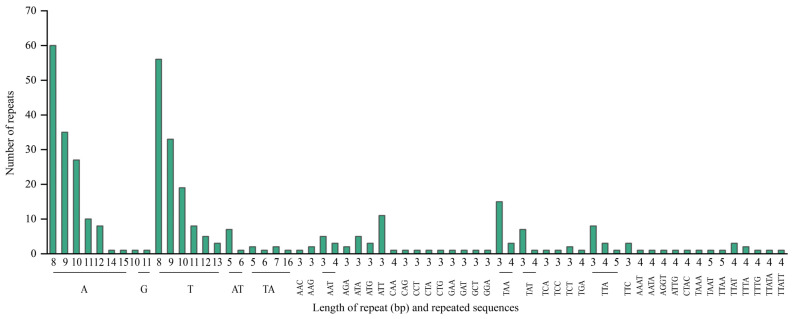
Numbers and types of SSR in the chloroplast genome of *R. giganteum*.

REPuter analysis identified 96 interspersed repeats in the chloroplast genome of *R. giganteum*, including 25 reverse, 32 palindromic, 28 forward, and 11 complementary repeats, ranging from 30 to 9841 bp in length (Figure 3). Additionally, 385 SSRs were detected, comprising 268 mononucleotide, 14 dinucleotide, 87 trinucleotide, and 16 tetranucleotide repeats (Figure 4). Mononucleotide repeats, predominantly A or T, were the most abundant.

### 3.4. IR Expansion and Contraction in Chloroplast Genomes

**Figure 5:**
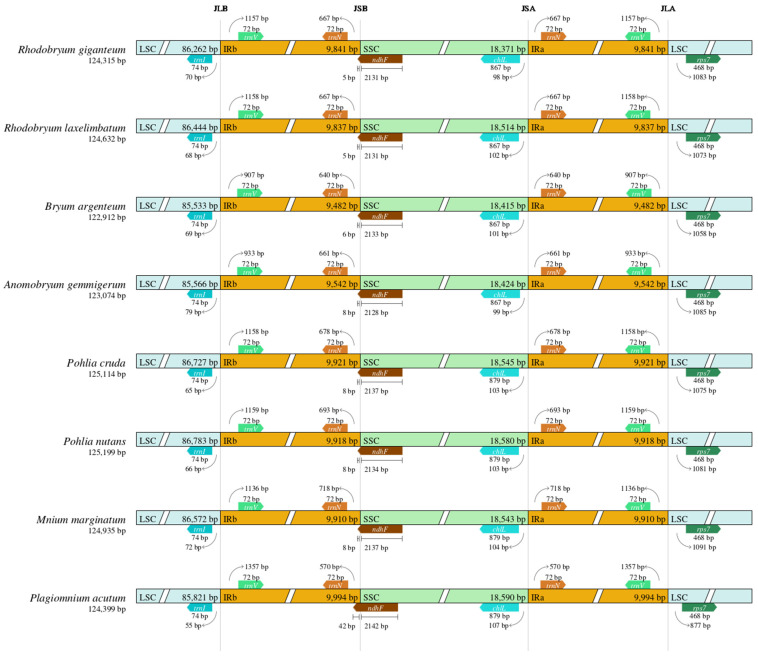
Comparison of the large single-copy (LSC) region, small single-copy (SSC) region, and inverted repeat (IR) junctions among the chloroplast genome sequences of 8 Bryales species.

IR/LSC and IR/SSC boundary regions of eight Bryales species were analyzed (Figure 5). Chloroplast genome lengths varied from 122,912 bp (*B. argenteum*) to 125,199 bp (*P. nutans*), with all species exhibiting similar tetrameric region boundary structures. Notably, the length from the *trnI* gene to the JLB region of *P. acutum* was reduced to 55 bp, while this distance ranged from 65 bp to 79 bp in the other seven species (*R. giganteum*, *R. laxelimbatum*, *P. cruda*, *P. nutans*, *M. marginatum*, *B. argenteum*, and *A. gemmigerum*). At the JSB boundary, all eight mosses had *ndhF* genes spanning both coding regions, with the *ndhF* gene of *P. acutum* being 42 bp in the IRb region, compared to 5 bp to 8 bp in the other mosses. Regarding the JLA boundary, the distances from the *trnV* genes of five mosses (*R. giganteum*, *R. laxelimbatum*, *P. cruda*, *P. nutans*, and *M. marginatum*) to the JLA junction ranged from 1136 bp to 1159 bp, while for *B. argenteum*, *A. gemmigerum*, and *P. acutum*, the distances were 907 bp, 933 bp, and 1357 bp, respectively. Despite these variations, the overall structure of the IR boundaries demonstrated high conservation, with no significant contraction or expansion observed across the studied species.

### 3.5. Comparison Analysis of the Chloroplast Genome Sequence of Bryales

**Figure 6:**
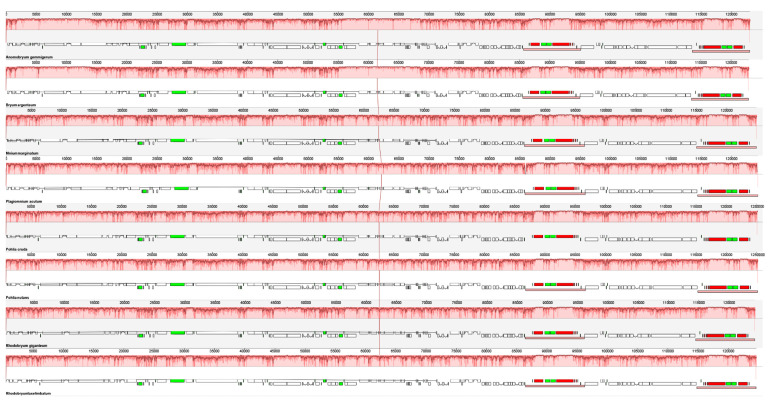
The Mauve alignment of 8 Bryales species. The alignment is illustrated with localized co-linear blocks, each represented by continuous colored regions. The boxes colored in white are annotated CDS, the boxes colored in green are tRNAs, and the blocks colored in red are rRNAs.

Co-linear analysis using MAUVE v2.4.0 software revealed high conservation in chloroplast genome structure and gene order among the eight Bryales species examined. The analysis showed no evident gene rearrangements or inversions, indicating a remarkable similarity in genome organization across these species (Figure 6). This conservation suggests a stable evolutionary history of chloroplast genomes within the Bryales order, potentially reflecting the importance of maintaining functional integrity in these organelles.

**Figure 7:**
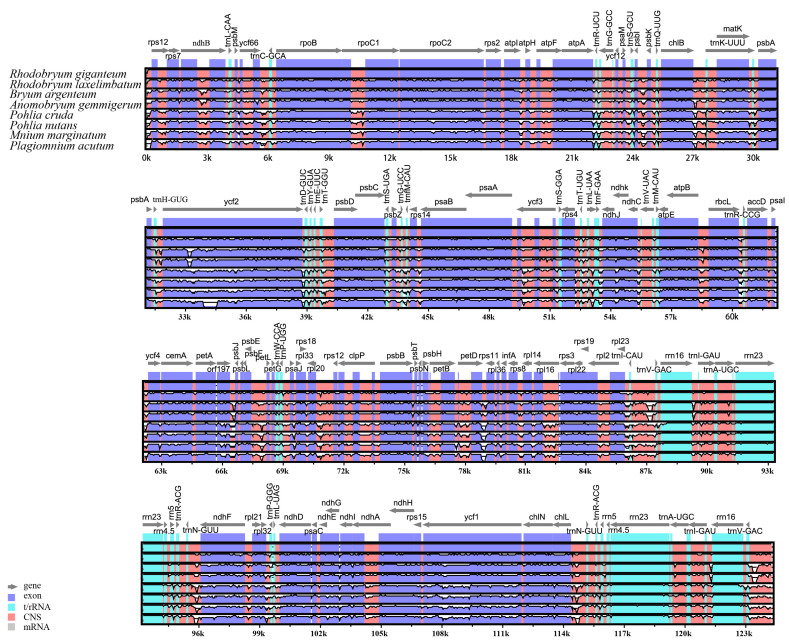
Sequence alignment plot for 8 Bryales species using mVISTA with *R. giganteum* chloroplast genome as a reference.

**Figure 8:**
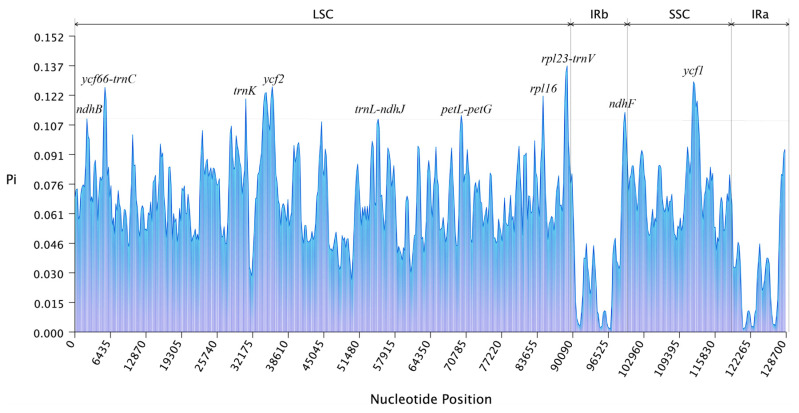
Nucleotide polymorphism analysis of the chloroplast genomes of Bryales species.

Genomic divergence and sequence identity among *R. giganteum* and seven other Bryales species were evaluated using mVISTA, with *R. giganteum*’s chloroplast genome as the reference sequence (Figure 7). Results revealed high sequence similarity across entire genomes, with coding regions showing greater conservation than non-coding regions. However, variant regions were identified within some coding sequences (e.g., *ndhB*, *ycf2*, and *ycf3*) and in intergenic spacer regions (e.g., *trnR*-*trnG*, *chlB*-*trnK*, *trnG*-*trnfM*, *psbE*-*petL*, *petD*-*rps11*, *trnI*-*trnV*, *trnN*-*ndhF*, and *chlL*-*trnN*). Nucleotide diversity (Pi) analysis across the chloroplast genomes (Figure 8) indicated higher variability in the LSC and SSC regions, particularly in *ndhB*, *ycf66*-*trnC*, *trnK*, *ycf2*, *trnL*-*ndhJ*, *petL*-*petG*, *rpl16*, *rpl23*-*trnV*, *ndhF*, and *ycf1*. These findings highlight specific genomic regions of evolutionary interest within Bryales chloroplast genomes, potentially reflecting differential selective pressures or functional constraints.

### 3.6. Selective Pressure Analysis of Chloroplast Genomes of Bryales Species

**Figure 9:**
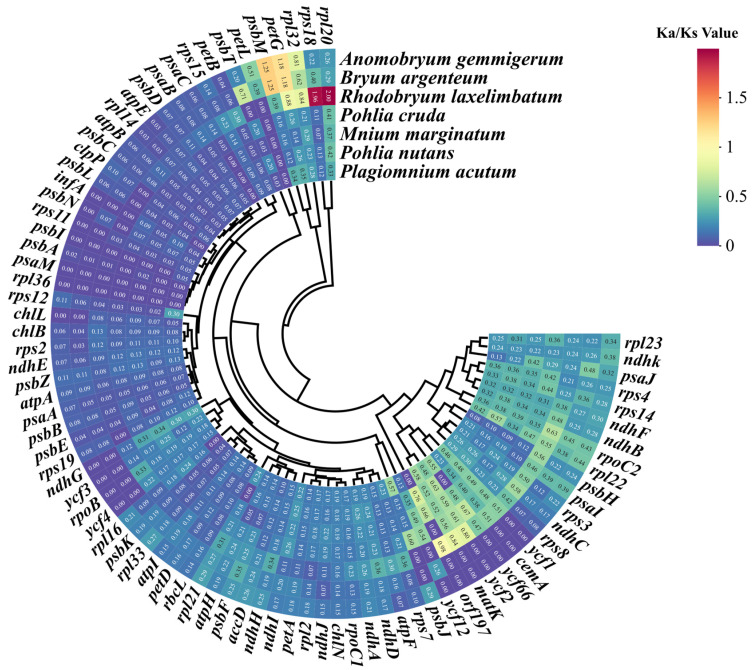
The Ka/Ks ratios of 83 protein-coding genes in the chloroplast genome of *R. giganteum* versus 7 other Bryales species.

Ka/Ks ratios were calculated for the *R. giganteum* chloroplast genome in comparison to seven other Bryales species (Figure 9). The average Ka/Ks ratio across 83 common protein-coding genes in the eight chloroplast genomes was 0.20. Notably, four genes (*rpl20*, *rps18*, *petG*, and *psbM*) exhibited Ka/Ks values exceeding 1, suggesting positive selection during evolution. Conversely, the remaining genes showed Ka/Ks ratios below 1, indicating strong purifying selection. These findings highlight differential evolutionary pressures on specific genes within Bryales chloroplast genomes, with most genes under conservative selection while a few key genes potentially undergoing adaptive evolution.

### 3.7. Phylogenetic Analysis

**Figure 10:**
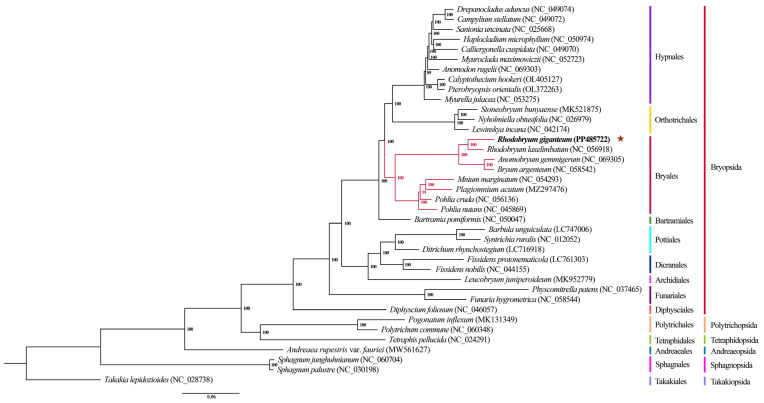
The ML tree constructed from chloroplast whole-genome sequences of 38 species. The red star represents the positions of *R. giganteum*. Branches are labeled with bootstrap (BS) support values.

A maximum-likelihood (ML) phylogenetic tree was constructed using complete chloroplast genome sequences to determine the phylogenetic position of *R. giganteum*, with *T. lepidozioides* as the outgroup (Figure 10). The results demonstrated strong support (100%) for the majority of nodes, with a few nodes receiving support of at least 89%. *Rhodobryum giganteum* was situated within the Bryales taxon, positioned between the Bartramiales and the Orthotrichales. The Bryales species formed a distinct monophyletic group, which was further divided into two sub-branches. One sub-branch consisted of *R. giganteum*, *R. laxelimbatum*, *A. gemmigerum*, and *B. argenteum*, while the other included *P. nutans*, *P. cruda*, *P. acutum*, and *M. marginatum*.

## 4. Discussion

Similar to most land plants, the genome displays a typical quadripartite structure and maintains a high level of conservation in terms of genome structure, gene content, and gene composition. The size of the *R. giganteum* chloroplast genome aligns with other Bryales members (124–125 kb) [43,44,45]. The structural composition and gene count in *R. giganteum* were consistent with those of other Bryales species, highlighting the overall conservatism of the plant chloroplast genome. GC content is a crucial feature influencing species distribution and environmental adaptability. The total GC content in the chloroplast genome of *R. giganteum* is 30.17%. Notably, there was a significant variation in GC content across different genome regions, with the IR region exhibiting higher GC content compared to the LSC and SSC regions, reflecting distinct structural and functional requirements of these genomic regions.

Codon usage bias in chloroplast genomes is primarily driven by natural selection and mutational pressure, with the third base of synonymous codons playing a crucial role [46]. Similar to other plant chloroplast genomes [47,48,49], the chloroplast genome of *R. giganteum* exhibits 29 codons with a high frequency of codon usage (RSCU > 1), all ending with A/U. In contrast, there are 33 codons with low codon usage frequency (RSCU < 1), most of which end with C/G. Plant chloroplast genomes commonly contain simple sequence repeats and long repetitive sequences [50], which are rich sources of genetic information and are considered mutation hotspots within genome sequences. These sequences serve as valuable molecular markers for studying species’ genetic evolution [51]. The chloroplast genome of *R. giganteum* displays abundant repetitive sequences, including forward repeats, palindromes, reverse repeats, and complementary repeats. Within SSRs, single nucleotide A/T repeat units are the most prevalent in the *R. giganteum* chloroplast genome, consistent with observations in most plant chloroplast genes [52,53]. The distribution of SSRs is mainly in the LSC and SSC regions, with fewer SSRs found in the IR regions. These repetitive sequences and SSRs in the *R. giganteum* chloroplast genome have important practical applications. They can be used as molecular markers for population genetic studies, phylogenetic analysis, and species identification. In conservation biology, these markers can help assess genetic diversity and inform protection strategies. Additionally, in medicinal plant research, they could potentially be used to identify and track strains with specific therapeutic properties [54].

Variations in chloroplast size are primarily due to the contraction or expansion of inverted repeat (IR) regions and single-copy regions (LSC and SSC) [55]. When comparing the IR boundaries in the chloroplast genome sequences of *R. giganteum* with the seven other chloroplast genome sequences, a high degree of conservation was observed in both the IR regions and single-copy regions of chloroplasts in Bryales (Figure 5). Furthermore, the highly variable regions identified through mVISTA analysis and nucleotide diversity analysis may provide more variable sites compared to standard DNA barcodes for plant taxonomy and phylogenetic analysis, thus potentially serving as molecular markers [56].

The Ka/Ks analysis is a crucial tool in molecular evolutionary studies that assesses selective pressures on gene sequences within the chloroplast genome. A ratio of Ka/Ks below 1 indicates purifying selection, a ratio above 1 points to positive selection, and a ratio of 1 signifies neutral drift [57]. In our analysis, only four genes (*rpl20*, *petG*, *rps18*, and *psbM*) showed ratios greater than 1, suggesting positive selection for adaptive mutations (Figure 9). Conversely, the majority of genes had Ka/Ks values below 1, indicating purifying selection. The genes under positive selection offer intriguing targets for investigating adaptive mechanisms in mosses, while most genes underscore the high conservation of functional integrity in these genes, aligning with the observed conservatism in the chloroplast genome.

The chloroplast genomes of 38 moss species were thoroughly analyzed using maximum likelihood (ML) to construct a phylogenetic tree of the Bryales. The analysis revealed that *R. giganteum* was grouped within Bryaceae alongside *R. laxelimbatum*, *A. gemmigerum*, and *B. argenteum*, with *R. giganteum* showing the closest relationship to *R. laxelimbatum*. At the class level, the phylogenetic placements of Takakiopsida, Sphagnopsida, Andreaeopsida, Tetraphidopsida, Polytrichopsida, and Bryopsida were consistent with previous studies [58,59]. The comprehensive analysis of whole chloroplast genomes proves to be crucial for studying moss phylogeny, as it offers higher accuracy compared to analyzing fragments. Therefore, the findings of this study will significantly contribute to the understanding of the evolutionary history of moss plants.

## 5. Conclusions

This study presents the first sequencing, assembly, and annotation of the complete chloroplast genome of *R. giganteum*, revealing a typical quadripartite structure spanning 124,315 bp and containing 128 genes. The analysis uncovered several key features, including a preference for A/U-ending codons, the presence of 96 repetitive sequences and 385 SSRs, and ten regions identified as high mutational hotspots. Notably, four genes (*rpl20*, *rps18*, *petG*, and *psbM*) showed signs of positive selection, suggesting their potential role in adaptive evolution. Phylogenetic analysis firmly placed *R. giganteum* within Bryales, closely related to *R. laxelimbatum*.

The genomic data presented in this study not only serve as a foundation for comparative analyses among mosses, potentially enriching our understanding of the evolutionary history of land plants, but also offer essential genetic resources for investigating the medicinal properties of *R. giganteum*. These resources may aid in conservation strategies and the development of novel therapeutic applications. Future studies could further explore functional genomics to elucidate the roles of specific genes, particularly those related to stress tolerance and secondary metabolite production in this important medicinal moss species.

## Figures and Tables

**Table 1:** The characteristics of *R. giganteum* chloroplast genome.

Category	Item	Describe
Chloroplast genome structure	Total length (bp)	124,315
	LSC length (bp)	86,261
	SSC length (bp)	18,371
	IRa/IRb length (bp)	9481
Gene composition	Gene number	128
	tRNA	37
	rRNA	8
	Protein-coding genes	83
GC content	Chloroplast gene	30.17%
	LSC	27.6%
	SSC	27.3%
	IRa/IRb	44.1%

## Data Availability

The *R. giganteum* chloroplast genome data sequenced in this study are deposited at NCBI (https://www.ncbi.nlm.nih.gov/nuccore, accessed on 29 June 2024) under accession number PP485722.

## Supplementary Materials

The following supporting information can be downloaded at: https://www.mdpi.com/article/10.3390/genes15070900/s1, Table S1. Genes in chloroplast genome of *R. giganteum*; Table S2. RSCU values and numbers for codons in the CDS analysis of the *R. giganteum* chloroplast genome.

## References

[1] Mohamed M.A.H. (1984). A synopsis of the genus *Rhodobryum* in Asia. J. Hattori Bot. Lab..

[2] Jia Y., He S. (2013). Species Catalogue of China. Plants: Bryophytes.

[3] Horwath A.B., Royles J., Tito R., Gudiño J.A., Allen N.S., Farfan-Rios W., Rapp J.M., Silman M.R., Malhi Y., Swamy V. (2019). Bryophyte stable isotope composition, diversity and biomass define tropical montane cloud forest extent. Proc. R. Soc. B.

[4] Singh S., Srivastava K., Gahtori D., Saxena D.K. (2017). Bryomonitoring of atmospheric elements in *Rhodobryum giganteum* (Schwaegr.) Par., growing in Uttarakhand Region of Indian Himalayas. Aerosol Air Qual. Res..

[5] Tan B.C. (1978). Book review. Bryologist.

[6] Wu P.C. (1977). *Rhodobryum giganteum* (Schwaegr.) Par can be used for curing cardiovascular disease. Acta Phytotaxon. Sin..

[7] National Compendium of Chinese Herbal Medicines Writing Group (1975). National Compendium of Chinese Herbal Medicine (Volume II).

[8] Jiangsu Institute of Botany (1991). Xinhua Compendium of Materia Medica (Volume III).

[9] Harris E.S. (2008). Ethnobryology: Traditional uses and folk classification of bryophytes. Bryologist.

[10] Asakawa Y. (2007). Biologically active compounds from bryophytes. Pure Appl. Chem..

[11] Bandyopadhyay A., Dey A. (2022). The ethno-medicinal and pharmaceutical attributes of bryophytes: A review. Phytomedicine Plus.

[12] Cai Y., Lu Y., Chen R., Wei Q., Lu X. (2011). Anti-hypoxia activity and related components of *Rhodobryum giganteum* Par. Phytomedicine.

[13] Li L., Zhao J. (2009). Determination of the volatile composition of *Rhodobryum giganteum* (Schwaegr.) Par. (Bryaceae) using solid-phase microextraction and gas chromatography/mass spectrometry (GC/MS). Molecules.

[14] Motti R., Palma A.D., de Falco B. (2023). Bryophytes used in folk medicine: An ethnobotanical overview. Horticulturae.

[15] Chevigny N., Schatz-Daas D., Lotfi F., Gualberto J.M. (2020). DNA repair and the stability of the plant mitochondrial genome. Int. J. Mol. Sci..

[16] Cao Z., Yang L., Xin Y., Xu W., Li Q., Zhang H., Tu Y., Song Y., Xin P. (2023). Comparative and phylogenetic analysis of complete chloroplast genomes from seven *Neocinnamomum* taxa (Lauraceae). Front. Plant Sci..

[17] Kousar M., Park J. (2023). Comparative analysis of the chloroplast genome of *Sicyos angulatus* with other seven species of Cucurbitaceae family. Genes.

[18] Li X., Yang Y., Henry R.J., Rossetto M., Wang Y., Chen S. (2015). Plant DNA barcoding: From gene to genome. Biol. Rev. Camb. Philos. Soc..

[19] Oliver M.J., Murdock A.G., Mishler B.D., Kuehl J.V., Boore J.L., Mandoli D.F., Everett K.D.E., Wolf P.G., Duffy A.M., Karol K.G. (2010). Chloroplast genome sequence of the moss *Tortula ruralis*: Gene content, polymorphism, and structural arrangement relative to other green plant chloroplast genomes. BMC Genom..

[20] Zhou X., Peng T., Zeng Y., Cai Y., Zuo Q., Zhang L., Dong S., Liu Y. (2023). Chromosome-level genome assembly of *Niphotrichum japonicum* provides new insights into heat stress responses in mosses. Front. Plant Sci..

[21] Chen S., Zhou Y., Chen Y., Gu J. (2018). Fastp: An ultra-fast all-in-one FASTQ preprocessor. Bioinformatics.

[22] Langmead B., Salzberg S.L. (2012). Fast gapped-read alignment with Bowtie 2. Nat. Methods.

[23] Prjibelski A., Antipov D., Meleshko D., Lapidus A., Korobeynikov A. (2020). Using SPAdes de novo assembler. Curr. Protoc. Bioinf..

[24] Boetzer M., Henkel C.V., Jansen H.J., Butler D., Pirovano W. (2011). Scaffolding pre-assembled contigs using SSPACE. Bioinformatics.

[25] Boetzer M., Pirovano W. (2012). Toward almost closed genomes with GapFiller. Genome Biol..

[26] Kearse M., Moir R., Wilson A., Stones-Havas S., Cheung M., Sturrock S., Buxton S., Cooper A., Markowitz S., Duran C. (2012). Geneious basic: An integrated and extendable desktop software platform for the organization and analysis of sequence data. Bioinformatics.

[27] Chan P.P., Lin B.Y., Mak A.J., Lowe T.M. (2021). tRNAscan-SE 2.0: Improved detection and functional classification of transfer RNA genes. Nucleic Acids Res..

[28] Greiner S., Lehwark P., Bock R. (2019). OrganellarGenomeDRAW (OGDRAW) version 1.3.1: Expanded toolkit for the graphical visualization of organellar genomes. Nucleic Acids Res..

[29] He B., Dong H., Jiang C., Cao F., Tao S., Xu L.A. (2016). Analysis of codon usage patterns in *Ginkgo biloba* reveals codon usage tendency from A/U-ending to G/C-ending. Sci. Rep..

[30] Wu Y., Zeng M.-Y., Wang H.-X., Lan S., Liu Z.-J., Zhang S., Li M.-H., Guan Y. (2024). The complete chloroplast genomes of *Bulbophyllum* (Orchidaceae) species: Insight into genome structure divergence and phylogenetic analysis. Int. J. Mol. Sci..

[31] Sharp P.M., Tuohy T.M.F., Mosurski K.R. (1986). Codon usage in yeast: Cluster analysis dearly differentiates highly and lowly expressed genes. Nucleic Acids Res..

[32] Beier S., Thiel T., Münch T., Scholz U., Mascher M. (2017). MISA-web: A web server for microsatellite prediction. Bioinformatics.

[33] Wang Y., Liang Q., Zhang C., Huang H., He H., Wang M., Li M., Huang Z., Tang Y., Chen Q. (2023). Sequencing and analysis of complete chloroplast genomes provide insight into the evolution and phylogeny of Chinese kale (*Brassica oleracea* var. *alboglabra*). Int. J. Mol. Sci.

[34] Kurtz S., Choudhuri J.V., Ohlebusch E., Schleiermacher C., Stoye J., Giegerich R. (2001). REPuter: The manifold applications of repeat analysis on a genomic scale. Nucleic Acids Res..

[35] Frazer K.A., Pachter L., Poliakov A., Rubin E.M., Dubchak I. (2004). VISTA: Computational tools for comparative genomics. Nucleic Acids Res..

[36] Li H., Guo Q., Xu L., Gao H., Liu L., Zhou X. (2023). CPJSdraw: Analysis and visualization of junction sites of chloroplast genomes. PeerJ.

[37] Rozas J., Ferrer-Mata A., Sánchez-DelBarrio J.C., Guirao-Rico S., Librado P., Ramos-Onsins S.E., Sánchez-Gracia A. (2017). DnaSP 6: DNA sequence polymorphism analysis of large data sets. Mol. Biol. Evol..

[38] Wang D., Zhang Y., Zhang Z., Zhu J., Yu J. (2010). KaKs_Calculator 2.0: A toolkit incorporating γ-series methods and sliding window strategies. Genom. Proteom. Bioinf..

[39] Ivanova Z., Sablok G., Daskalova E., Zahmanova G., Apostolova E., Yahubyan G., Baev V. (2017). Chloroplast genome analysis of resurrection tertiary relict *Haberlea rhodopensis* highlights genes important for desiccation stress response. Front. Plant Sci..

[40] Katoh K., Standley D.M. (2013). MAFFT multiple sequence alignment software version 7: Improvements in performance and usability. Mol. Biol. Evol..

[41] Kalyaanamoorthy S., Minh B.Q., Wong T.K.F., von Haeseler A., Jermiin L.S. (2017). ModelFinder: Fast model selection for accurate phylogenetic estimates. Nat. Methods.

[42] Minh B.Q., Schmidt H., Chernomor O., Schrempf D., Woodhams M., Haeseler A.V., Lanfear R. (2020). IQ-TREE 2: New models and efficient methods for phylogenetic inference in the genomic era. Mol. Biol. Evol..

[43] Shi S., Li S., Zhang S., Shen F., Niu J., Li L., Zhao J. (2021). The complete chloroplast genome of *Mnium marginatum* (With.) P. Beauv. Mitochondrial DNA Part B.

[44] Liu S., Fang S., Cong B., Li T., Yi D., Zhang Z., Zhao L., Zhang P. (2022). The antarctic moss *Pohlia nutans* genome provides insights into the evolution of bryophytes and the adaptation to extreme terrestrial habitats. Front. Plant Sci..

[45] Li S., Shen F., Zhang S., Niu J., Niu Y., Li L., Zhao J. (2021). The complete chloroplast genome of *Rhodobryum laxelimbatum* (Hampe ex Ochi) Z. Iwatsuki and T. J. Koponen. Mitochondrial DNA Part B.

[46] Zeng Y., Shen L., Chen S., Qu S., Hou N. (2023). Codon usage profiling of chloroplast genome in Juglandaceae. Forests.

[47] Zhang H., Huang T., Zhou Q., Sheng Q., Zhu Z. (2023). Complete chloroplast genomes and phylogenetic relationships of *Bougainvillea spectabilis* and *Bougainvillea glabra* (Nyctaginaceae). Int. J. Mol. Sci..

[48] Zhu H., Liu J., Li H., Yue C., Gao M. (2023). Complete chloroplast genome structural characterization and comparative analysis of *Viburnum japonicum* (Adoxaceae). Forests.

[49] Sablok G., Nayak K.C., Vazquez F., Tatarinova T.V. (2011). Synonymous codon usage, GC_3_, and evolutionary patterns across plastomes of three pooid model species: Emerging grass genome models for monocots. Mol. Biotechnol..

[50] Qin Z., Wang Y., Wang Q., Li A., Hou F., Zhang L. (2015). Evolution analysis of simple sequence repeats in plant genome. PLoS ONE.

[51] Yang T., Aishan S., Zhu J., Qin Y., Liu J., Liu H., Tie J., Wang J., Qin R. (2023). Chloroplast genomes and phylogenetic analysis of three *Carthamus* (Asteraceae) species. Int. J. Mol. Sci..

[52] Lin X., Lee S.Y., Ni J., Zhang X., Hu X., Zou P., Wang W., Liu G. (2023). Comparative analyses of chloroplast genome provide effective molecular markers for species and cultivar identification in *Bougainvillea*. Int. J. Mol. Sci..

[53] Alawfi M.S., Alzahrani D.A., Albokhari E.J. (2023). Complete chloroplast genome sequences of two *Ehretia* trees (*Ehretia cymosa* and *Ehretia obtusifolia*): Genome structures and phylogenetic analysis. Forests.

[54] Xu J., Wang Y., Wu K., Chen J. (2024). Identification and characterization of functionally relevant SSR markers in natural *Dalbergia odorifera* populations. BMC Plant Biol..

[55] dePamphilis C.W., Palmer J.D. (1990). Loss of photosynthetic and chlororespiratory genes from the plastid genome of a parasitic flowering plant. Nature.

[56] Chen J., Wang F., Zhao Z., Li M., Liu Z., Peng D. (2023). Complete chloroplast genomes and comparative analyses of three *Paraphalaenopsis* (Aeridinae, Orchidaceae) species. Int. J. Mol. Sci..

[57] Yang Z., Nielsen R. (2000). Estimating synonymous and nonsynonymous substitution rates under realistic evolutionary models. Mol. Biol. Evol..

[58] Cox C.J., Goffinet B., Shaw A.J., Boles S.B. (2004). Phylogenetic relationships among the mosses based on heterogeneous Bayesian analysis of multiple genes from multiple genomic compartments. Syst. Bot..

[59] Bell D., Lin Q., Gerelle W.K., Joya S., Chang Y., Taylor Z.N., Rothfels C.J., Larsson A., Villarreal J.C., Li F.W. (2020). Organellomic data sets confirm a cryptic consensus on (unrooted) land-plant relationships and provide new insights into bryophyte molecular evolution. Am. J. Bot..

