# Supplementary material for: The Complete Chloroplast Genome Sequence of the Medicinal Moss Rhodobryum giganteum (Bryaceae, Bryophyta): Comparative Genomics and Phylogenetic Analyses
Source: Genes (Basel). 2024 Jul 10;15(7):900. doi: 10.3390/genes15070900 (PMC11275618; doi:10.3390/genes15070900)
Supplement: Supplementary file 1 [file genes-15-00900-s001.zip › genes-3080861-supplementary.pdf]

Table S1. Genes in chloroplast genome of *R. giganteum*.

Category	Gene group	Gene name
Photosynthesis	Subunits of photosystem I	*psaA*, *psaB*, *psaC*, *psaI*, *psaJ*, *psaM*
	Subunits of photosystem II	*psbA*, *psbB*, *psbC*, *psbD*, *psbE*, *psbF*, *psbH*, *psbI*, *psbJ*, *psbK*, *psbL*, *psbM*, *psbN*, *psbT*, *psbZ*
	Subunits of NADH dehydrogenase	*ndhA* *, *ndhB* *, *ndhC*, *ndhD*, *ndhE*, *ndhF*, *ndhG*, *ndhH*, *ndhI*, *ndhJ*, *ndhk*
	Subunits of cytochrome b/f complex	*petA*, *petB* *, *petD* *, *petG*, *petL*
	Subunits of ATP synthase	*atpA*, *atpB*, *atpE*, *atpF* *, *atpH*, *atpI*
	Large subunit of rubisco	*rbcL*
	Subunits photochlorophyllide reductase	*chlB*, *chlL*, *chlN*
Self-replication	Proteins of large ribosomal subunit	*rpl14*, *rpl16* *, *rpl2* *, *rpl20*, *rpl21*, *rpl22*, *rpl23*, *rpl32*, *rpl33*, *rpl36*
	Proteins of small ribosomal subunit	*rps11*, *rps12* [a], *, *rps14*, *rps15*, *rps18*, *rps19*, *rps2*, *rps3*, *rps4*, *rps7*, *rps8*
	Subunits of RNA polymerase	*rpoB*, *rpoC1* *, *rpoC2*
	Ribosomal RNAs	*rrn16* (2), *rrn23* (2), *rrn4.5* (2), *rrn5* (2)
	Transfer RNAs	*trnA-UGC* * (2), *trnC-GCA*, *trnD-GUC*, *trnE-UUC*, *trnF-GAA*, *trnG-GCC**, *trnG-UCC*, *trnH-GUG*, *trnI-CAU*, *trnI-GAU* * (2), *trnK-UUU* *, *trnL-CAA*, *trnL-UAA* *, *trnL-UAG*, *trnM-CAU*, *trnN-GUU* (2), *trnP-GGG*, *trnP-UGG*, *trnQ-UUG*, *trnR-ACG* (2), *trnR-CCG*, *trnR-UCU*, *trnS-GCU*, *trnS-GGA*, *trnS-UGA*, *trnT-GGU*, *trnT-UGU*, *trnV-GAC* (2), *trnV-UAC* *, *trnW-CCA*, *trnY-GUA*, *trnfM-CAU*
Other genes	Maturase	*matK*
	Protease	*clpP* **
	Envelope membrane protein	*cemA*
	Acetyl-CoA carboxylase	*accD*
	Translation initiation factor	*infA*
	Component of TIC complex	*ycf1*
	Component of 2-MD heteromeric AAA-ATPase complex	*ycf2*
Genes with unknown function	Hypothetical chloroplast reading frames	*ycf12*, *ycf3* **, *ycf4*, *ycf66* *
	ORF	*orf197*

Gene *, gene containing a single intron; Gene **, gene containing two introns; Gene[a], trans-splicing genes; Gene (2), number of copies of two genes.

Table S2. RSCU values and numbers for codons in the CDS analysis of the *R. giganteum* chloroplast genome.

Amino Acid	Codon	Number	RSCU	Amino Acid	Codon	Number	RSCU
Ter	UAA	44	2.5385	Met	AUG	429	1
Ter	UAG	5	0.2885	Asn	AAC	141	0.2342
Ter	UGA	3	0.1731	Asn	AAU	1063	1.7658
Ala	GCA	378	1.3322	Pro	CCA	264	1.3732
Ala	GCC	105	0.37	Pro	CCC	90	0.4681
Ala	GCG	64	0.2256	Pro	CCG	44	0.2289
Ala	GCU	588	2.0722	Pro	CCU	371	1.9298
Cys	UGC	32	0.3556	Gln	CAA	682	1.7532
Cys	UGU	148	1.6444	Gln	CAG	96	0.2468
Asp	GAC	72	0.2028	Arg	AGA	295	1.8342
Asp	GAU	638	1.7972	Arg	AGG	25	0.1554
Glu	GAA	933	1.8152	Arg	CGA	215	1.3368
Glu	GAG	95	0.1848	Arg	CGC	67	0.4166
Phe	UUC	219	0.3341	Arg	CGG	45	0.2798
Phe	UUU	1092	1.6659	Arg	CGU	318	1.9772
Gly	GGA	536	1.7276	Ser	AGC	58	0.244
Gly	GGC	98	0.3159	Ser	AGU	304	1.2791
Gly	GGG	76	0.245	Ser	UCA	359	1.5105
Gly	GGU	531	1.7115	Ser	UCC	96	0.4039
His	CAC	59	0.2921	Ser	UCG	80	0.3366
His	CAU	345	1.7079	Ser	UCU	529	2.2258
Ile	AUA	604	0.9562	Thr	ACA	345	1.411
Ile	AUC	172	0.2723	Thr	ACC	109	0.4458
Ile	AUU	1119	1.7715	Thr	ACG	53	0.2168
Lys	AAA	1429	1.8344	Thr	ACU	471	1.9264
Lys	AAG	129	0.1656	Val	GUA	404	1.6456
Leu	CUA	175	0.4834	Val	GUC	68	0.277
Leu	CUC	48	0.1326	Val	GUG	76	0.3096
Leu	CUG	27	0.0746	Val	GUU	434	1.7678
Leu	CUU	409	1.1298	Trp	UGG	387	1
Leu	UUA	1300	3.5912	Tyr	UAC	99	0.2409
Leu	UUG	213	0.5884	Tyr	UAU	723	1.7591
